# Supplementary material for: Effects of Temperature on Transparent Exopolymer Particle Production and Organic Carbon Allocation of Four Marine Phytoplankton Species
Source: Biology (Basel). 2022 Jul 14;11(7):1056. doi: 10.3390/biology11071056 (PMC9312018; doi:10.3390/biology11071056)
Supplement: Supplementary file 1 [file biology-11-01056-s001.zip › biology-1755581-supplementary.pdf]

Supplementary material

Table S1. Specific growth rates of the four phytoplankton species.

Temperature (℃)	Growth rate (d^{-1})			
	P. micans	*C. affinis*	*N. closterium*	*S. trichoidea*
16	0.184±0.029**	0.506±0.02	0.413±0.011	0.107±0.004
20	0.261±0.015	0.532±0.007	0.705±0.014	0.118±0.007
24	0.291±0.014	0.524±0.008	0.993±0.01**	0.123±0.007

**：P<0.05

Table S2. TEP concentration of *S. trichoidea*

Temperature	TEP conc.per liter (μg Xeq./L)	TEP conc.per Chl *a* (μg Xeq./L)
16℃	1325.18±101.94	8.2015±1.12
20℃	1427.11±134.85	7.78647±0.58
24℃	1868.84±77.86**	7.34563±0.19

**: P<0.05
